# Distribution and genomic variation of ammonia-oxidizing archaea in abyssal and hadal surface sediments

**DOI:** 10.1038/s43705-023-00341-6

**Published:** 2023-12-22

**Authors:** Blandine Trouche, Clemens Schauberger, Feriel Bouderka, Jean-Christophe Auguet, Caroline Belser, Julie Poulain, Bo Thamdrup, Patrick Wincker, Sophie Arnaud-Haond, Ronnie N. Glud, Loïs Maignien

**Affiliations:** 1 Univ Brest, CNRS, Ifremer, UMR6197 Biologie et Ecologie des Ecosystèmes marins Profonds, F-29280 Plouzané, France; 2 Hadal & Nordcee, Department of Biology, University of Southern Denmark, Odense, Denmark; 3 MARBEC, Univ Montpellier, Ifremer, IRD, CNRS, Sète, France; 4 Génomique Métabolique, Genoscope, Institut François Jacob, CEA, CNRS, University of Évry, Université Paris-Saclay, 91057 Evry, France; 5 Department of Ocean and Environmental Sciences, Tokyo University of Marine Science and Technology, Tokyo, Japan; 6 Danish Institute for Advanced Study (DIAS), University of Southern Denmark, Campusvej 55, 5230 Odense, Denmark; 7 Marine Biological Laboratory, Josephine Bay Paul Center for Comparative Molecular Biology and Evolution, Woods Hole, MA USA

**Keywords:** Archaeal genomics, Metagenomics, Microbial ecology, Biogeography

## Abstract

Ammonia-oxidizing archaea of the phylum Thaumarchaeota play a central role in the biogeochemical cycling of nitrogen in benthic sediments, at the interface between pelagic and subsurface ecosystems. However, our understanding of their niche separation and of the processes controlling their population structure in hadal and abyssal surface sediments is still limited. Here, we reconstructed 47 AOA metagenome-assembled genomes (MAGs) from surface sediments of the Atacama and Kermadec trench systems. They formed deep-sea-specific groups within the family *Nitrosopumilaceae* and were assigned to six *amo*A gene-based clades. MAGs from different clades had distinct distribution patterns along oxygen-ammonium counter gradients in surface sediments. At the species level, MAGs thus seemed to form different ecotypes and follow deterministic niche-based distributions. In contrast, intraspecific population structure, defined by patterns of Single Nucleotide Variants (SNV), seemed to reflect more complex contributions of both deterministic and stochastic processes. Firstly, the bathymetric range had a strong effect on population structure, with distinct populations in abyssal plains and hadal trenches. Then, hadal populations were clearly separated by trench system, suggesting a strong isolation-by-topography effect, whereas abyssal populations were rather controlled by sediment depth or geographic distances, depending on the clade considered. Interestingly, genetic variability between samples was lowest in sediment layers where the mean MAG coverage was highest, highlighting the importance of selective pressure linked with each AOA clade’s ecological niche. Overall, our results show that deep-sea AOA genome distributions seem to follow both deterministic and stochastic processes, depending on the genomic variability scale considered.

## Introduction

Ammonia-oxidizing archaea (AOA) of the phylum Thaumarchaeota are widespread and abundant members of the ocean realm [1–3]. They are key contributors to the carbon and nitrogen cycles, as they are generally able to conserve energy from the aerobic oxidation of ammonia to nitrite, the first and limiting step in the nitrification process [4–6], a role first evidenced by the isolation of *Nitrosopumilus maritimus* SCM1 from the gravel (sediment) of a marine tropical fish tank [5]. Marine AOA belong to order Nitrosopumilales (NP), recently reclassified as family *Nitrosopumilaceae* in SILVA db v138.1 [7] and GTDB v207 [8, 9]. They form distinct clades, as delineated by *amo*A (ammonia monooxygenase subunit A) gene sequences [10], present in the water column and sediments [10, 11]. Hadopelagic waters of North Pacific trench systems are populated by clades *amo*A-NP-alpha and *amo*A-NP-gamma [12–15]. The *amo*A-NP-gamma clade, containing type strain *Nitrosopumilus maritimus* SCM1, seems to be widespread in oceans, irrespective of water depth, while *amo*A-NP-alpha populations appear most abundant in bathy- and abyssopelagic environments (1000–6000 m) [12, 15, 16]. By contrast, the dominant clades reported in sediments of abyssal plains are *amo*A-NP-theta and *amo*A-NP-delta [16, 17]; however, data are scarce regarding AOA distribution in the deep sea, let alone in hadal sediments.

Studying benthic AOA along the abyssal-hadal depth gradient is of particular interest since these sediments are known to have very different microbial activity levels [18, 19], with generally higher activity in trenches due to increased accumulation of organic matter in the trenches by topographical funneling and mass-wasting events [20–22]. This results in different potential habitats for AOA due to variations in oxygen penetration depths between hadal sediments, where oxygen is depleted after a few centimeters [19, 23], and abyssal sediments, where it persists decimeters down [19, 24]. In addition to this difference in substrate influx and oxic zone thickness, hadal trenches are distinguishable from adjacent abyssal plains by the increased hydrostatic pressure, and their specific morphology that could contribute to biogeographic isolation.

Along this line, previous microbial diversity studies in Pacific trench systems have shown a remarkable similarity of hadobenthic communities along trench axes and between trench systems, but strong dissimilarity with their respective adjacent abyssal sites [25–27]. In the sediments of the Atacama and Kermadec trenches, sediment depth, in conjunction with redox stratification, remains the dominant structuring factor [27], in line with the strong stratification with sediment depth commonly reported for marine microbial communities [28–30], supporting the deterministic influence of environmental conditions in these systems.

Recently, reconstruction of metagenome-assembled genomes provided the opportunity to inspect finer-grained variations in genomic content in longitudinal or time series [31–34], a major step toward a better appraisal of the relative contribution of stochastic and deterministic processes on microbial population genomics. For example, while surface ocean cosmopolitan SAR11 populations were shown to be shaped by environmental selection [33], subseafloor crustal fluid populations seem to be mainly structured by the stochastic forces of dispersal and drift [32]. Hwang and Girguis [34] leveraged similar tools to highlight different strategies of genetic diversification between ocean basins for the ubiquitous pelagic AOA lineage “*Candidatus* Nitrosopelagicus brevis”. However, population structure at the genomic level and the relative contribution of selection and drift have rarely been investigated in strongly stratified environments such as marine surface sediments, even less so at abyssal and hadal depths.

Previous studies have already demonstrated that different AOA clades adopt distinct ecological strategies, resulting in clear niche partitioning between different lineages, mostly in relation to the oxygen and ammonium concentration gradients and to pH [35–39]. AOA are thus ideal candidates to further investigate the relative contribution of additional environmental parameters such as geographic distance, water and sediment depth to their distribution, from lineage level to finer genomic variations. In this study, we characterized the diversity of AOA in abyssal and hadal sediments at the genome and at the population level to gain insights into the niche separation of these archaea and discuss the ecological and evolutionary processes likely at play. Building on a study of the general microbiome of the Atacama and Kermadec trenches in the South Pacific Ocean and their adjacent abyssal plains [27], we leveraged 56 metagenomes generated from the same samples to study the distribution of Thaumarchaeota clades in these sediments at higher taxonomic and genetic resolution.

## Material and methods

### Sampling sites and slicing scheme

Samples were collected during two cruises in November 2017 and March 2018 as part of the HADES-ERC project. Two sites were sampled during the first cruise targeting the Kermadec trench, north of New Zealand, one in the trench axis using a multicorer (K6, 9555 m below sea level (mbsl)), and the other on the adjacent subducting plate using a boxcorer (K7, 6080 mbsl). The second set of samples originated from the Atacama trench, off the coast of Chile, with two trench axis sites (A10 and A3, 7770 and 7915 mbsl) and two sites located on the adjacent abyssal plain (A7, 5500 mbsl) or continental margin (A9, 4050 mbsl). All samples from the Atacama trench were collected using a multicorer. Triplicate cores were recovered for Atacama trench axis site A3 and the adjacent abyssal plain site A7. A map of the sampling sites is available in supplementary (Supplementary Fig. S1) and in Schauberger et al. [40] and Glud et al. [19].

The recovered sediment cores were sliced onboard immediately upon recovery into depth layers following a standard scheme (0–1, 1–3, 3–5, 5–10, 10–15, and 15–30 cm), as described in Trouche et al. [41]. In total, 60 samples were obtained from 10 cores. Details of the samples and sites can be found in Supplementary Table S1.

Sediment layers were classified as part of the oxic, nitrogenous, or ferruginous zones based on in situ oxygen profiles from the same locations [19], and the penetration depth of nitrate measured in parallel on board [42], as described in Schauberger et al. [27].

### DNA extraction, library construction and sequencing

DNA extractions were performed in a sterile shore lab, using approximately 10 g of sediment with the PowerMax Soil DNA Isolation Kit according to the manufacturer’s instructions (MO BIO Laboratories, Inc.; Qiagen, Hilden, Germany) with modifications: the elution buffer was left on the spin filter membrane for 10 min at room temperature before centrifugation in order to increase DNA yield. Each of the resulting 5-mL DNA solutions was stored at –80 °C.

Metagenomic libraries were prepared from 10 ng or less of DNA with the NEBNext Ultra II DNA Library prep kit (New England Biolabs, MA, USA). After quantification and quality control, library concentrations were normalized to 10 nM and applied to cluster generation according to the Illumina Cbot User Guide (Part #15006165). Sequencing of libraries was performed according to the Novaseq 6000 System User Guide Part #20023471 (Illumina, San Diego, CA, USA) in paired-end mode (2 × 150 bp). See Supplementary Material for the details of library preparation and sequencing carried out at Génoscope (Evry, France).

### Assembly and binning

The quality filtration of the demultiplexed metagenomic raw reads was carried out with Illumina-Utils python scripts [43] following recommendations by Minoche et al. [44]. Metagenomes were then split into ten co-assembly groups based on de novo comparison of the unassembled metagenomes using *k*-mer counts with Simka [45]. Composition of the co-assembly groups can be found in Supplementary Table S2. Most of the following steps were performed with the help of the Snakemake workflows [46] available with Anvi’o v7 [47, 48].

We co-assembled the samples using Megahit (v1.1 [49]) with preset meta-sensitive and minimum contig length of 1000 bp. Identification of Open Reading Frames (ORFs) in the contigs was run with Prodigal [50] and functional annotation obtained using KOfamscan [51], the COG (2020 release [52]) and arCOG databases [53].

After mapping of the short reads on the resulting contigs with bowtie2 [54, 55], automatic binning was performed with Concoct [56], restricting the number of bins to half the number of predicted bacterial genomes to prevent fragmentation errors. Archaeal bins were then inspected and successively refined manually twice using Anvi’o’s interactive interface [57]. Completeness and redundancy were estimated by Anvi’o based on the archaeal single-copy core gene collection [58].

Reconstructed Metagenome-Assembled Genomes (MAGs) were dereplicated based on pyANI with a minimum alignment fraction of 0.5, and a similarity threshold of 0.95. They were then once again run through the mapping steps of the workflow to obtain final coverage values. When detection (or breadth of coverage) was over 0.7, mean coverage values were used as a proxy for the relative abundance of the MAG; in other cases, it was set to 0. The lineage workflow from CheckM was applied to evaluate MAG quality (v1.1.3 [59]). We finally assigned MAG quality categories based on recommendations in the Genome Standards Consortium [60].

### External reference MAGs

To add context to our results, we included MAGs from other deep-sea studies to the phylogenetic and phylogenomic sections of our analysis. In particular, we retrieved 4 MAGs reconstructed by Zhong et al. [15] from the water column of the Mariana Trench (MTA1, MTA4, MTA5, MTA6), 9 MAGs reconstructed by Kerou et al. [17] from marine sediments at abyssal depths (NPMR_NP_delta_1 to 3, NPMR_NP_theta_1 to 5 and NPMR_NP_iota_1) and 22 MAGs reconstructed by Zhou et al. [61] from sediments of Challenger’s Deep in the Mariana Trench and adjacent slope sites (Supplementary Table S3). They were run through the same steps as described above for gene calling and functional annotation.

### Phylogenetic placement of *amo*A genes

To identify the clades of ammonia-oxidizing archaea present in our metagenomes and MAGs, we extracted the sequences annotated as ammonia monooxygenase subunit A (*amo*A) by KOfam from our co-assemblies and from the external MAGs. We dereplicated them using CD-Hit [62] with 100% global sequence identity (100% overlap of the shorter sequence). We used a blastn search against the non-redundant NCBI nucleotide collection to confirm gene assignment and determine domain-level taxonomy [63]. We then aligned the sequences matching archaeal *amo*A genes using MAFFT with default parameters (v7.273 [64]) and placed them in the reference tree by Alves et al. [10] using EPA-ng [65]. We visualized this tree in R (v3.6.1) using packages ggtree and treeio [66, 67].

### Phylogenomic placement and taxonomic annotation of MAGs

Taxonomic assignment of the MAGs was performed using GTDB-tk v1.7.0 *classify_wf* workflow [68] with the GTDB database (r202 [8, 69]). We used the concatenated alignment of 118 markers generated by the GTDB-tk *align* command, after masking of positions with over 0.5 gap frequency, to reconstruct a maximum likelihood tree in IQTREE (v2.0.3 [70, 71]) under the model WAG with 1000 ultrafast bootstrap replicates.

This method was used to generate a tree containing MAGs from this study, together with external reference MAGs and GTDB representative sequences for the Nitrososphaerales order (Nitrosopumilales in the accepted taxonomy). The tree was rooted by choosing genus *Pyrococcus* as an outgroup. We applied the same method to generate a tree of only MAGs from this study. We visualized these trees in R (v3.6.1) using packages ggtree and treeio [66, 67].

Clade identification was performed as described above based on *amo*A genes [10]. When the *amo*A gene was absent from the reconstructed genome, MAG cladistic classification was inferred from the phylogenomic tree.

### Single nucleotide and single amino acid variant analyses

During the mapping of the short reads on our MAG cluster representatives, we performed single nucleotide variants (SNVs) calling to investigate genomic variability using the *anvi_profile* function from Anvi’o, with flags --skip-SNV-profiling = false, and --profile-SCVs = true. We then used command *anvi-gen-variability-profile* with engine NT (nucleotide) to compute tables listing all variable positions in samples where the MAG had a coverage over 10x. In addition, we required reported positions to have a coverage above 10× in every sample considered and a minimum departure from consensus of 0.1, departure from consensus being computed as the total number of reads not matching the consensus divided by the total number of mapped reads. Given these selection criteria, this analysis was done only for the four main clades for MAGs with coverage over 10× in at least 40 samples when possible or 20 samples for the abyssal clades. The MAG with coverage over 10× in the highest number of samples was chosen for illustration purposes for each of the main clades.

We used these nucleotide variability tables to estimate distances between metagenomes by computing the fixation index (F_ST_) according to Schloissnig et al. [72] with function *anvi-gen-fixation-index-matrix*, and determined a hierarchical clustering based on this index (*anvi-matrix-to-newick*). We then introduced the resulting tables and trees in R (v3.6.1) for the subsequent statistical analyses and visualization, which was done using packages aplot, dplyr [73], ggplot2 [74], ggtree [66], patchwork [75], tidyr [76], tidytree [77], treeio [67] and vegan [78]. The fully reproducible workflow for this analysis is available at https://loimai.github.io/abyssMG/.

## Results

### Reconstruction of AOA MAGs

The final dataset was composed of 56 metagenomic libraries with a mean of 159,868,532 raw reads (min 116,086,397, max 259,670,853). Sequencing failed for four libraries, corresponding to the deepest sediment layers of abyssal site A7’s triplicate cores. On average, 96.6% of reads passed the quality filter and were input to the co-assembly (Supplementary Table S2).

We reconstructed a total of 47 *Nitrosopumilaceae* MAGs (taxonomy based on the Genome Taxonomy Database (GTDB), accepted taxonomy order Nitrosopumilales). Despite high sequencing depths, only two of these MAGs were of high quality, with completeness over 90% and redundancy under 5% (Supplementary Table S4). Eight other MAGs had completeness over 70% and redundancy under 7%. The remaining MAGs had lower completeness estimates, down to 28%, and redundancy below 10% with a mean of 3.48% except for two outliers (12.62% for A7D_Bin_00083 and 14.98% for A7D_Bin_00161) (Supplementary Table S4). The genome size of the ten MAGs over 70% complete varied between 0.91 and 1.3 Mbp, in accordance with previous reports of marine free-living Thaumarchaeota [79]. Mean GC content among MAGs was 33.4% (Supplementary Table S4).

**Fig. 1:**
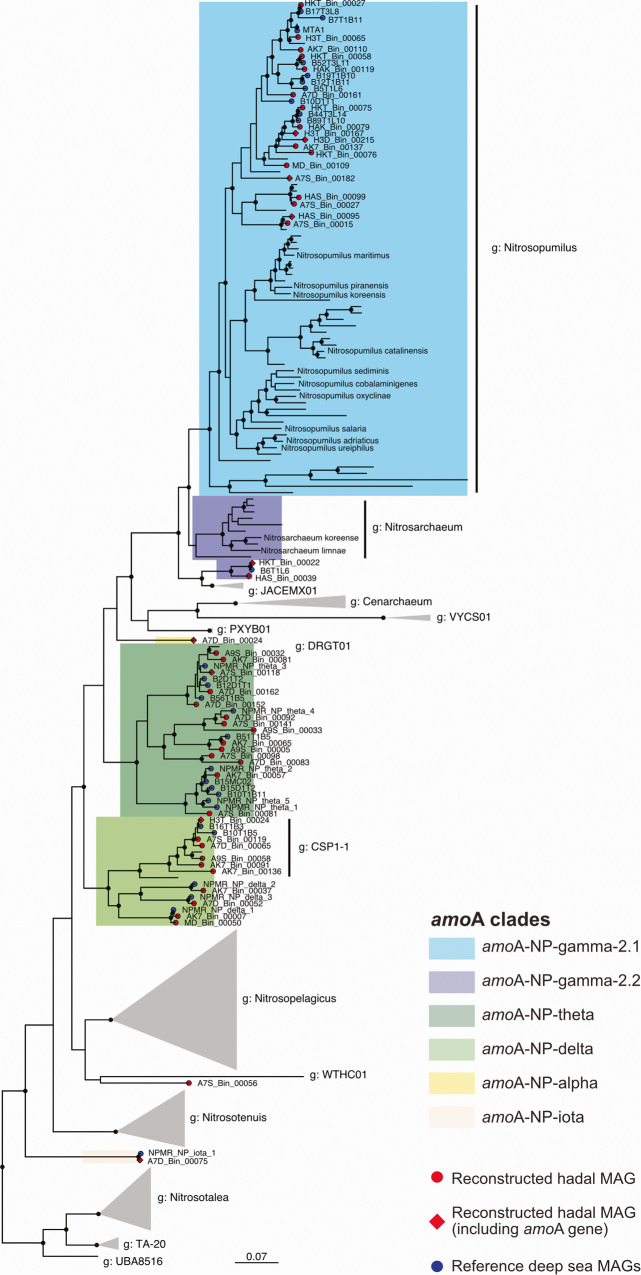
Phylogenomic tree of family *Nitrosopumilaceae* (GTDB taxonomy, order Nitrosopumilales in accepted taxonomy). This tree was subsetted from a phylogenomic tree of order Nitrososphaerales generated with GTDB r202, rooted using genus *Pyrococcus* as an outgroup (see Material and Methods for details). Nodes with Ultrafast bootstrap (1000 iterations) support >95% were marked in black solid points. The 47 MAGs presented in this study are shown with red diamonds or circles, depending on whether the *amo*A gene was identified or not, respectively. Blue circles correspond to 35 reference MAGs from three other deep-sea studies added to provide context to our results [15, 17, 61]. Colored shading indicates *amo*A-NP-clades for the groups of interest based on Alves et al. [10].

Our co-assemblies contained *amo*A genes belonging to 7 *amo*A-NP clades, namely *amo*A-NP- gamma-2.1, -gamma.2.2, -gamma-3, -alpha, -delta, -theta and -iota (Supplementary Fig. S2). Among our reconstructed MAGs, 10 contained *amo*A genes that were affiliated with six of these clades, excepting *amo*A-NP-gamma-3 (Supplementary Fig. S2). Eighteen MAGs were placed in genus *Nitrosopumilus* (*amo*A-NP-gamma-2.1) but were not closely related to the cultivated or enriched strains (Fig. 1). The two *amo*A-NP-gamma-2.2 MAGs (HAS_Bin_00039 and HKT_Bin_00022) also clustered apart from the representative *Nitrosarchaeum* genomes belonging to the same clade (Fig. 1). Ten MAGs from our study clustered together with the amoA-NP-delta MAGs reconstructed by Kerou et al. [17], two MAGs from Zhou et al. [61] and two GTDB representative genomes of genus *CSP1-1*. Fourteen MAGs formed a cluster with one GTDB representative genome of a genus named *DRGT01* and all Kerou et al. [17] amoA-NP-theta MAGs. Finally, three additional MAGs were sole representatives of their clades, with one *amo*A-NP-alpha MAG (A7D_Bin_00024), one *amo*A-NP-iota (A7D_Bin_00075) clustering with the MAG of the same clade reconstructed by Kerou et al. [17], and one unassigned MAG (A7S_Bin_00056).

### Distribution of the AOA MAGs

**Fig. 2:**
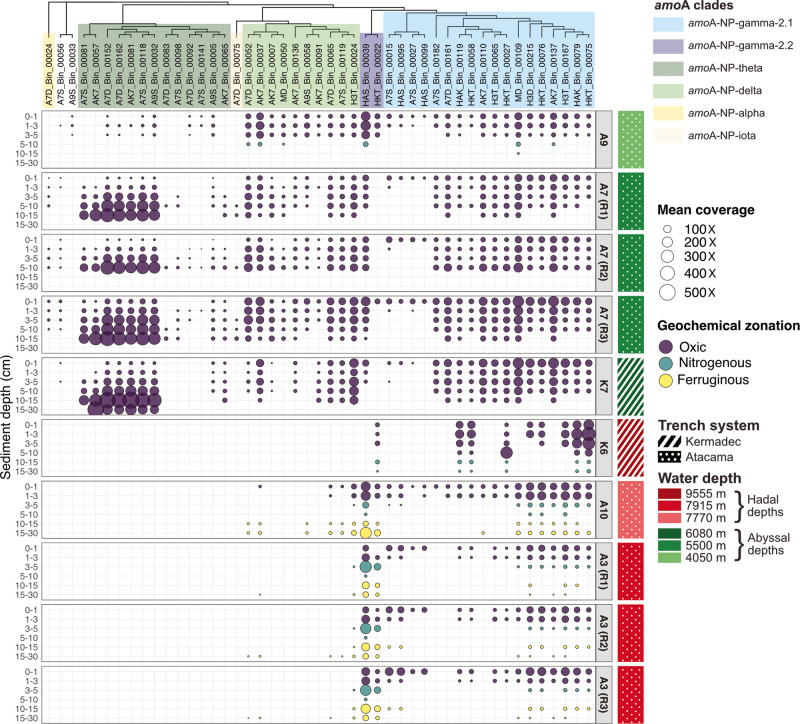
Mean coverage, used here as a proxy for relative abundance, of AOA MAGs in the samples they were reconstructed from. The size of the points is proportional to the mean coverage of the considered MAG in a given sample. Mean coverage was set to 0 in samples where detection (or breadth of coverage) was below 0.7. Bubbles are colored according to geochemical zonation of the sample (characterized in Schauberger et al. [27]). Samples are grouped by site and replicate core when appropriate (color code on the right-hand side of the figure), and ordered vertically by increasing horizon depth. The top panel represents the ML phylogenomic tree of the reconstructed MAGs generated using GTDB toolkit and IQTREE, with *amo*A clades colored according to Alves et al. [10] and the results from Fig. 1.

During the final mapping step of the workflow, between 0.36 and 5.27% of reads mapped to the AOA MAGs across samples (average 2.19%). We observed the presence of AOA in oxic horizons of all sites, with members of the *amo*A-NP-theta, -delta and -gamma-2.1 and 2.2 clades dominating the samples (Fig. 2). *amo*A-NP-gamma-2.1 MAGs were detected in the oxic surface sediments of all sites, generally decreasing in coverage with increasing sediment depth. *amo*A-NP-gamma-2.2 MAGs followed a similar trend, except that they had low or no coverage at both hadal and abyssal Kermadec sites K6 and K7, and at abyssal site A7 for HKT_Bin_00022. At both hadal sites A3 and A10, they were detected at relatively high coverage throughout the sediment column except between 5 and 10 cm, but including surrounding nitrogenous and ferruginous layers.

*amo*A-NP-delta and *amo*A-NP-theta were nearly exclusively abyssal clades. *amo*A-NP-delta MAGs were most present in upper to mid horizons of all abyssal sites, but some MAGs also recruited reads in the deeper sediment layers of site A10. *amo*A-NP-theta were only found at open ocean abyssal sites, increasing in coverage with sediment depth, though some populations were fairly rare and others dominated the deep horizons of sites A7 and K7, possibly highlighting distinct clusters. Finally, single representatives of clades *amo*A-NP-alpha and *amo*A-NP-iota were restricted to very specific sediment layers of abyssal site A7: surface and deeper layers, respectively.

### Population-level biogeography of AOA clades

**Fig. 3:**
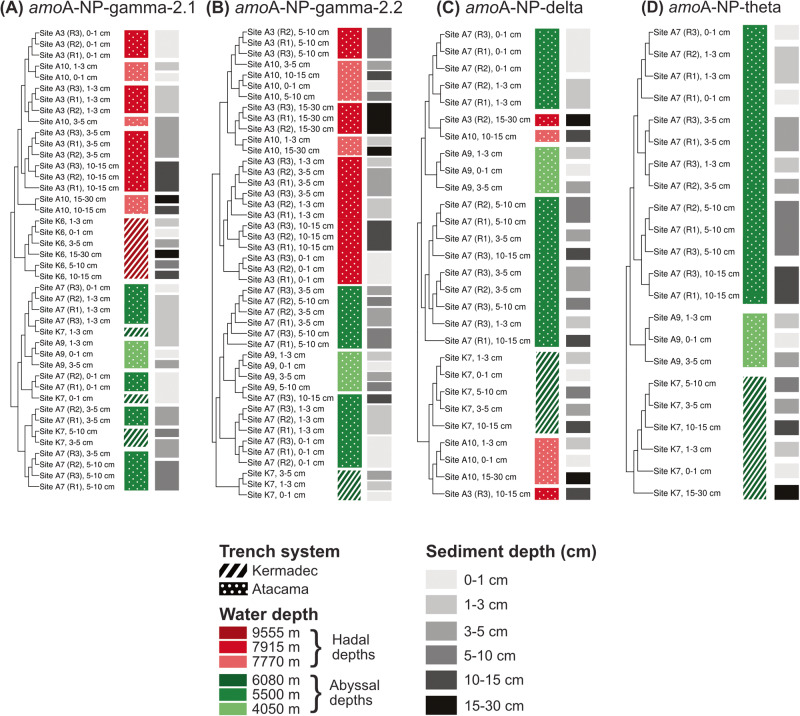
Hierarchical clustering of samples based on the fixation index (F_ST_) computed using nucleotide-level variability profiles for clade representatives. **A** MAG HAK_Bin_00079, **B** MAG HAS_Bin_00039, **C** MAG H3T_Bin_00024, and **D** MAG A9S_Bin_00032. Hierarchical clustering based on ward linkage.

We then compared MAG population structure based on SNV patterns by computing F_ST_ indices as a measure of individual MAG genetic dissimilarity between samples. In general, sample clustering patterns were conserved within each clade and only results for single-clade representatives are shown (Fig. 3, see Supplementary Figs. S3–S6 for results based on other MAGs).

Variability profile comparisons of clade *amo*A-NP-gamma-2.1 led to a first-order separation of samples according to ocean realm (abyssal vs hadal, PERMANOVA test *p* = 0.02) (Fig. 3A). In the hadal subset, samples clustered based on trench system (Kermadec vs. Atacama, Tukey HSD, *p* = 0.02) and Atacama trench axis samples (A3 and A10 sites) grouping showed the influence of both sampling site and sediment horizon (PERMANOVA test *p* = 0.001). In contrast, we did not observe a separation of samples originating from the Atacama and Kermadec regions in the abyssal subcluster, and these rather clustered according to the sediment layer, with an upper group (0 to 3–5 cm), and a lower group (3–5 to 15 cm) (Fig. 3A).

The observations were slightly different for clade *amo*A-NP-gamma-2.2 representative MAG (Fig. 3B). Separation between hadal and abyssal samples remained (Tukey HSD, *p* = 0.0082), but with no clear aggregation pattern at hadal depth apart from the replicate samples, though both site and horizon gave significant results to the PERMANOVA test (*p* < 0.006). The low coverage of this MAG in Kermadec hadal samples (K6) prevented a comparison between trench axes. In contrast to *amo*A-NP-gamma-2.1, samples originating from different abyssal sites clustered apart (PERMANOVA test *p* < 0.002). A similar clustering of Kermadec abyssal samples was detected for clades *amo*A-NP-delta and -theta (Fig. 3C, D, PERMANOVA test *p* = 0.001 and 0.03 respectively). When *amo*A-NP-delta-affiliated MAGs were sufficiently covered in hadal samples, as illustrated in Fig. 3C, we observed an intriguing pattern of clustering of some of the deeper hadal samples with the upper layers of the Atacama abyssal sites. Finally, the *amo*A-NP-theta clade was the only one for which clustering seemed to be primarily linked with the sampling site (PERMANOVA test *p* = 0.03).

In addition to these observations, we noted that replicate samples for sites A3 and A7 mostly clustered together for all clades. For all MAGs examined, there was also a clear link between clustering patterns based on F_ST_ and sediment depth, although this effect was nested within the wider environmental site characteristics (Fig. 3A, B) and will be further explored by focusing on site A7.

### Evolution of genetic variability of AOA with burial in the sediments

The influence of burial on AOA genome microdiversity during the sedimentation process can be difficult to disentangle from other environmental factors when considering multiple sampling stations. We therefore investigated changes in population structure down triplicate sediment cores from abyssal site A7, where biogeochemical conditions were identical and where all clades were present and persisted up to 10 cm below the seafloor. As before, we present results for one representative MAG for each clade but supplementary figures are provided (Supplementary Figs. S7–S9).

**Fig. 4:**
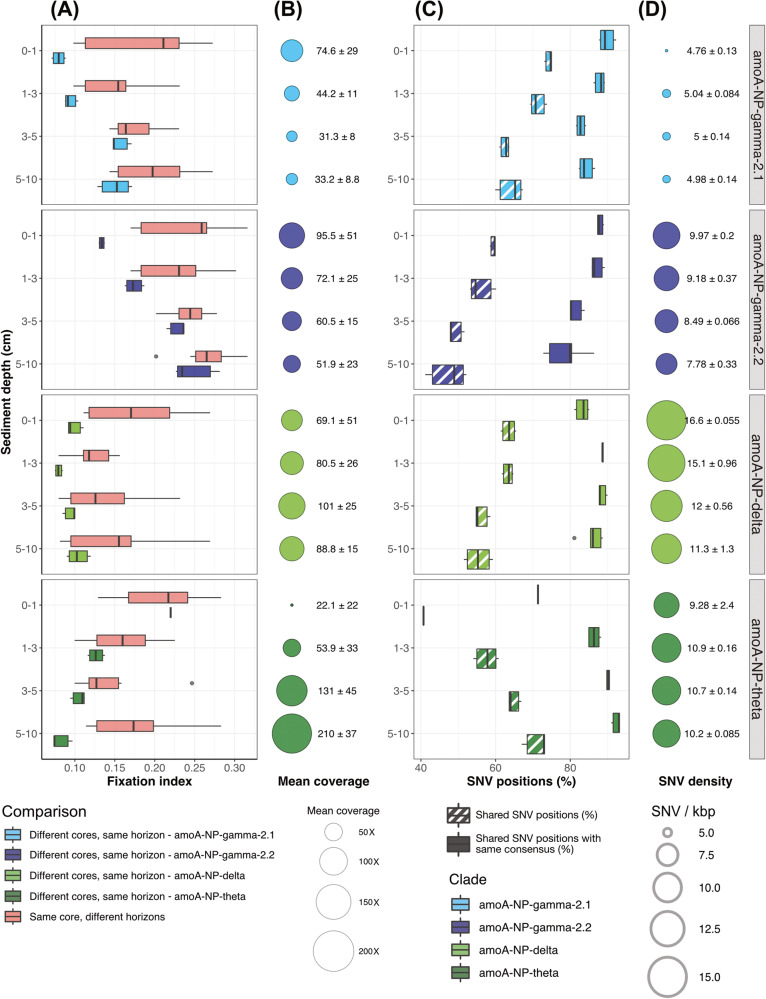
Analysis of downcore genetic variability at abyssal site A7. **A** Downcore comparison of fixation index (F_ST_) values between core triplicates of abyssal site A7, for each of the four main clades. Comparison between the same sediment layers from different cores is colored according to MAG *amo*A clade and comparison between different horizons of the same core in red. **B** MAG mean coverage in each sediment layer of the triplicate A7 cores, illustrated by circle size and clarified as mean ± standard deviation. **C** Percentage of single nucleotide variants (SNV) identified as variable positions in both samples when comparing similar sediment layers originating from different cores, and percentage of these shared SNVs presenting the same consensus nucleotide. **D** Mean SNV density in each sediment layer, summarized as mean ± standard deviation. MAGs considered for this analysis are the same as in Fig. 3: HAK_Bin_00079 for clade *amo*A-NP-gamma-2.1, HAS_Bin_00039 for clade *amo*A-NP-gamma-2.2, H3T_Bin_00024 for clade *amo*A-NP-delta, and A9S_Bin_00032 for clade *amo*A-NP-theta.

When comparing the genetic distance for each MAG between samples of the same sediment layer in replicate cores (hereafter inter-core comparison) and the distance between samples of the same core at different sediment depth (intra-core comparison), we observed that the mean inter-core distance was overall significantly lower than the mean intra-core distance (Kruskal–Wallis rank sum test, *p* < 5.8e^–4^) (Fig. 4A).

Though this pattern was general, the evolution of mean inter-core distance with sediment depth differed between clades. Indeed, it generally increased between the uppermost and lowest layers surveyed for clades *amo*A-NP-gamma-2.1 and -gamma-2.2, was overall stable for *amo*A-NP-delta, and decreased for *amo*A-NP-theta. More precisely, the lowest inter-core distances seemed to be reached at or near sediment layers where the considered MAG had the highest coverage: 0–1 cm for *amo*A-NP-gamma-2.1 and amoA-NP-gamma-2.2, 1–3 cm for amoA-NP-delta and 5–10 cm for amoA-NP-theta (Fig. 4A, B).

Based on the definition of the fixation index [72], a lower value can be attributed to either a lower variability between populations of the compared samples, and/or a higher within-sample variability. To distinguish between these possible influences, we further investigated the genetic variability of our MAGs in similar sediment layers of different cores (Fig. 4C, D).

The percentage of shared SNV positions, including those presenting the same consensus nucleotide (Fig. 4C), followed an opposite trend to the inter-core fixation index values for clades *amo*A-NP-gamma-2.1, -gamma-2.2 and -theta, and to a lesser extent *amo*A-NP-delta. In fact, fitting a linear regression to the evolution of the fixation index against the shared SNV, and shared SNV with the same consensus fractions yielded adjusted *R*^2^ between 0.81 and 0.97 with *p* values < 9.931e^–10^ for the first three clades (Supplementary Table S5). For *amo*A-NP-delta’s representative MAG, the linear regression was less well fitted (adjusted R^2^ 0.439 and 0.3647, *p* < 1e^–3^). On the other hand, downcore trends in SNV density evolution were not significant for clades *amo*A-NP-gamma-2.1 and *amo*A-NP-theta (Fig. 4D). For *amo*A-NP-gamma-2.2 and -delta, the difference in median was weakly significant (Kruskal–Wallis rank sum test, *p* = 0.015 and 0.024 respectively). In both cases, SNV density declined with sediment depth. Here raw SNV density values were considered since the application of a stringent filter of coverage during SNV discovery prevented differences in coverage to influence SNV detection rates. The highest proportion of shared SNV positions was reached by MAG HAK_Bin_00079 of clade *amo*A-NP-gamma-2.1 in the first sediment layer (75.04%). This MAG also had the lowest amount of SNVs detected, with a mean of 4.76 SNVs/kbp in horizon 0–1 cm (Fig. 4D). Overall, the strong correlation between fixation index and shared SNV fraction seemed to primarily link the observed F_ST_ patterns with variations in between-sample variability.

## Discussion

Our working hypotheses motivating this study were, firstly, that hadal sediments would harbor distinct AOA clades adapted to these extreme depths, secondly, that large geographic distances between our sampling sites (~8000 km) would lead to distinct AOA distribution following a typical distance decay relationship observed elsewhere [41], and thirdly that the topology of hadal trenches may strongly limit dispersion, favoring genetic drift and leading to distinct distribution in different trenches. Our results highlight the importance of exploring different genomic diversity scales from species to populations in order to reveal ecological and evolutionary processes controlling AOA distribution in deep-sea sediments.

### Deep-sea-specific AOA clades are differently distributed along sedimentary redox gradients in abyssal and trench systems

Both shallow and hadopelagic waters are typically dominated by *amo*A-NP-gamma-2.1 AOA, comprising genus *Nitrosopumilus* [13, 15]. Here, we reconstructed 18 *amo*A-NP-gamma-2.1 MAGs. Though they were indeed placed in genus *Nitrosopumilus* (Fig. 1), they were not closely related to cultivated or enriched strains such as *Nitrosopumilus maritimus* [5], which were recovered from shallower environments [80–83]. They rather formed a deep-sea cluster, grouping together 18 MAGs from this study, 10 of the chosen reference deep-sea MAGs, and 8 GTDB MAGs also originating from deep-sea samples [14, 84–89]. Interestingly, some of these latter MAGs were deep-sea sponge-associated *Nitrosopumilaceae*, while others were free-living organisms, suggesting a stronger influence of water depth than free-living or host-associated lifestyle in separating this cluster from the shallower water cluster. Similarly, the recovered MAGs affiliated with typically abyssobenthic clades *amo*A-NP-theta and *amo*A-NP-delta clustered remarkably close to external reference MAGs from abyssal plains [17] and Challenger’s Deep adjacent slopes [61] in our phylogenomic tree (Fig. 1), with very few representatives present in the GTDB [90–92].

Clades *amo*A-NP-gamma-2.1 and -gamma-2.2 were found to have a ubiquitous distribution in the upper layers of the sampling sites (Fig. 2). Though it is unclear how related pelagic and benthic *amo*A-NP-gamma-2.1 and -gamma-2.2 AOA are, their distribution pattern seemed to indicate a wide dispersal capability, most likely through the water column. The unexpected “resurgence” in coverage for *amo*A-NP-gamma-2.2 clade members in the ferruginous zone of trench axis sites A10 and A3 was puzzling. Nitric oxide dismutation (NO) has recently been proposed as a source of oxygen for AOA in anoxic environments [93]; however, geochemical characterization of the same study sites showed that both nitrate and nitrite, necessary for the reaction, are depleted at these depths [42]. Reactive iron and manganese oxides, which could have been alternative electron acceptors, were also shown to be depleted in these layers. Hence, as of now, the energy metabolism of these microorganisms remains enigmatic.

In contrast with the *amo*A-NP-gamma clades, it seemed that *amo*A-NP-delta and -theta clades were strictly benthic clades, with the highest coverage observed here in different abyssal sediment layers (0–10 and 3–30 cm, respectively) (Fig. 2). This observation was in agreement with previous studies that showed that *amo*A-NP-theta *Nitrosopumilaceae* dominated subsurface AOA communities, while *amo*A-NP-delta exhibited little variation in abundance with sediment depth [16, 17, 24]. The *amo*A-NP-theta clade could be adapted to low-oxygen environments, since they are absent from hadal sediments where the oxic zone is much shallower and the low-oxygen niche could be absent [19]. Alternatively, they could have developed an affinity for low organic reactivity environments, another feature of abyssal sediments. As for the *amo*A-NP-delta clade, their presence at abyssal site A9 where oxygen levels are more comparable to those of hadal sites [19] discounts the low-oxygen hypothesis. They could be more sensitive to hydrostatic pressure and thus less capable of transitioning to the hadal zone, given that they have often been identified in estuarine environments [94, 95]. In any case, the presence of the same *amo*A-NP-delta MAGs on both sides of the Atacama trench at sites A7 and A9 but not in the trench axis hinted at a deterministic environmental influence rather than a lack of dispersal (Fig. 2).

Overall, our results show that abyssal and hadal sediments are populated by AOA specific to these deep-sea environments, and more particularly to different niches related to the redox gradient. It thus appears that lineage-level AOA distribution in the deep-sea follows a classical niche theory [96, 97], mostly controlled by environmental parameters. Consequently, AOA distribution may be strongly coupled with organic matter input to the seafloor, which depends on ocean surface primary productivity, oceanic depth, trench morphology and substrate funneling, etc.

### Relative contribution of geographic distance and environmental constraints to AOA genome- and population-level distribution

To gain further insights into the drivers of deep-sea sedimentary AOA distribution at finer evolutionary scales, we investigated their population-level biogeography by characterizing their genetic variability using within-MAG patterns of point mutations (SNVs). In contrast to clade and genome level patterns, we obtained strong evidence for water depth influence on population structure. For ubiquitous clades *amo*A-NP-gamma-2.1 and -gamma-2.2, F_ST_-based clustering patterns delineated distinct populations along the bathymetric gradient, with a clear separation of abyssal from hadal samples (Fig. 3A, B). As was observed by Schauberger et al. [27] with a metabarcoding dataset on these samples, oceanic depth and the parameters associated with the transition between abyssal and hadal realms had a stronger structuring influence than geographic distance or differences in primary productivity above both trenches. Results regarding *amo*A-NP-delta and theta were less conclusive due to the lack of coverage of these MAGs in hadal samples.

In addition to water depth, our results highlighted the imprint of geographic distance on genomic variability and population structure at abyssal depths. We indeed observed a separation of samples according to their sampling region for *amo*A-NP-gamma-2.2 and *amo*A-NP-delta and *amo*A-NP-theta in the abyssal cluster (Fig. 3B–D). Here we did not have the appropriate metadata to disentangle between potential stochastic processes (dispersal limitation, drift, etc.) and environmental filtering. However, this observation was not repeated for *amo*A-NP-gamma-2.1 in abyssal samples: populations found in similar sediment layers on both sides of the Atacama trench axis or separated by thousands of kilometers were more closely related than with populations found a few vertical centimeters apart in the same sediment core (Fig. 3A). Different processes may explain this distinct behavior: on the one hand, ecological niches of *amo*A-NP-gamma AOA are generally located close to the sediment surface or in overlying waters, a position that may favor their dispersal in the water column compared to their more deeply entrenched counterparts. On the other hand, a strong purifying selection may filter for a small subset of pelagic populations able to adopt a benthic lifestyle. The relatively low SNV density observed in *amo*A-NP-gamma-2.1 (mean SNV density by sample 4.5 SNVs/kbp, compared to 8.7, 7.1 and 12.5 SNVs/kbp for *amo*A-NP-gamma-2.2, -delta and -theta, respectively) would argue for the latter explanation. The high dispersal capability and low genetic variability of clade *amo*A-NP-gamma-2.1 featured here across considerable geographic distance make it an interesting target to study the connectivity between benthic and pelagic populations in the future.

Finally, the potential for hadal trenches to act as a barrier to dispersal could only be evaluated with the MAGs from the *amo*A-NP-gamma-2.1 clade that had enough coverage in both trench systems. This clade clearly exhibited a clustering by trench, with separated grouping of Kermadec and Atacama trench axis samples (Fig. 3A). Such segregation may result from differences in sediment composition between trenches, by dispersal limitations leading to population-level ecological drift, or by the combination of both.

In addition, for all clades, the link between fixation index and sediment depth was clear but appeared nested within other influences (such as for the Atacama abyssal samples for clade *amo*A-NP-delta, Fig. 3C). This observation was in line with general observations of strong stratification of microbial communities with sediment depth in marine environments in general [28–30] and in the Atacama trench in particular [27]. Interestingly, our results suggested that niche structure, which determines the preferential sediment depth of the different AOA clades, is strongly correlated with their respective dispersal capacities, which in turn determines population structure. This was well illustrated by results from *amo*A-NP-gamma-2.1 members (Fig. 3A and Supplementary Fig. S3), generally living closer to the sediment surface and exhibiting long-range genomic homogeneity, while deeply entrenched *amo*A-NP-theta tended to display site-specific population structure (Fig. 3D and Supplementary Fig. S6).

### Vertical population structuring processes depend on ecological niche

Based on these latter observations, we specifically investigated the effect of sediment depth on AOA population structure. We thus focused our population analysis on triplicate cores taken from two multicorer casts, meters apart, at Atacama abyssal site A7. Previous studies have shown that geochemical gradients along sediment depth exert a strong and deterministic selection on community structure [27, 98–100]. We thus hypothesized that a similar pattern could emerge at the population level.

In our study, fixation indices between identical sediment layers in different cores were consistently lower than between different sediment layers in the same core (Fig. 4A). Hence, similarly to community assembly, population assembly seemed strongly controlled by downcore biogeochemistry rather than stochastic factors. In addition, this inter-core horizontal comparison was consistently at its lowest for sediment depths best fitting the niche of each clade (i.e., where coverage was highest): close to the surface for *amo*A-NP-gammas, between 3 and 10 cm for *amo*A-NP-delta, and below 5 cm for *amo*A-NP-theta. The highest proportions of shared SNVs and shared SNVs with the same consensus matched these F_ST_ minima (Fig. 4C). Together, these results support the hypothesis of a specific ecological niche for each clade where genetic variability is submitted to strong selective pressures.

This is most visible for the *amo*A-NP-theta clade, with the lowest fixation index values between samples of sediment layer 5–10 cm (Fig. 4A). Though we see a limited increase in SNV density with sediment depth that could suggest random within-sample diversification, it is actually associated with a strong increase in the number of shared variable positions between samples and variable positions with the same consensus nucleotide (Fig. 4C). This strongly supports the influence of convergent selection. Contrastingly, for *amo*A-NP-gamma-2.1, -gamma-2.2 and -delta, we observed an increase in fixation index with increasing sediment depth (below 3 cm for *amo*A-NP-delta) (Fig. 4A), suggesting populations drifting apart with burial in the sediments either as a result of dispersal limitation or due to the decay of selective pressure while departing from the clade’s optimum niche. These results illustrate the usefulness of metagenomic data in growing our understanding of microbial biogeography at multiple genomic scales.

## Conclusion

Here, we used metagenomic data to reconstruct 10 good-quality *Nitrosopumilaceae* MAGs, and 37 additional bins of varying completeness. We showed that at the genome level, they fall within deep-sea-specific clades, the distribution of which corresponds to distinct ecological niches: the predominance of *amo*A-NP-gamma clades in the surface layers of all sites contrasted with the positioning of *amo*A-NP-theta and delta in the deeper sediments of open ocean abyssal sites where oxygen persists. We also confirmed the strong structuring influence of the transition between hadal and abyssal realms, even at the genomic level. In the future, it will be particularly interesting to delve deeper into this genomic variability and the potential processes explaining its specificity to one or the other realm.

When investigating the downcore genetic variability of the reconstructed genomes, we found hints of the influence of purifying selective pressure in the layers best fitting the ecological niche (i.e., where genomes had the highest coverage), particularly visible for *amo*A-NP-theta. In other layers apparently offering less favorable conditions for life, signatures of stochastic processes such as drift were detected, as exemplified by *amo*A-NP-gamma clades below 3 cm. These findings build upon previous studies highlighting the vertical deterministic structuring of microbial communities in marine sediments [98–100] and demonstrate that stochastic processes, of increasing importance in deeper sediment layers and in the subseafloor [32], are already at play in the oxic zone of comparably young sediments.

### Supplementary information

Supplementary Information

Table S1

Table S2

Table S3

Table S4

Table S6

## Data Availability

The dataset generated for this study has been submitted to the European Nucleotide Archive (ENA) under project PRJEB57745 as part of the larger eDNAbyss project (PRJEB39225). Details of the sample correspondence are provided in Supplementary Table S2. Assembled MAGs are available on ENA under study PRJEB60556. In addition, annotated contigs and profile databases, as well as scripts for the full bioinformatic workflow will be available upon publication at https://loimai.github.io/abyssMG/.
