# Supplementary material for: Distribution and genomic variation of ammonia-oxidizing archaea in abyssal and hadal surface sediments
Source: ISME Commun. 2023 Dec 22;3:133. doi: 10.1038/s43705-023-00341-6 (PMC10746724; doi:10.1038/s43705-023-00341-6)
Supplement: Supplementary file 1 — Supplementary Information [file 43705_2023_341_MOESM1_ESM.pdf]

Supplementary material for Trouche *et al.*, Distribution and genomic variation of ammonia-oxidizing archaea in abyssal and hadal surface sediments

Supplementary information:

<u>Metagenomic library preparation and sequencing</u>

1. <u>Sequencing library preparation</u>

According to the relatively low DNA quantities extracted, 10 ng or less of genomic DNA were sonicated and the NEBNext Ultra II DNA Library prep kit for Illumina was manually applied. Fragments were end-repaired, 3'-adenylated and NEXTflex DNA barcoded adaptors were added by using NEBNext Ultra II DNA Library prep kit for Illumina (New England Biolabs, MA, USA). After two consecutive 1x AMPure clean ups, the ligated products were PCR-amplified with NEBNext® Ultra II Q5 Master Mix included in the kit, followed by 0.8x AMPure XP purification.

2. <u>Sequencing library quality control</u>

All libraries were quantified first by Quant-it dsDNA HS using a Fluoroskan Ascent instrument (ThermoFisher Scientific, Waltham, MA, USA) then by qPCR with the KAPA Library Quantification Kit for Illumina Libraries (Kapa Biosystems, Wilmington, MA, USA) on an MXPro instrument (Agilent Technologies, Santa Clara, CA, USA). Library profiles were assessed using a high throughput microfluidic capillary electrophoresis system (LabChip GX, Perkin Elmer, Waltham, MA).

3. <u>Sequencing procedures</u>

Library concentrations were normalized to 10 nM by addition of Tris-Cl 10 mM (pH 8.5) and applied to cluster generation according to the Illumina Cbot User Guide (Part # 15006165). Sequencing of libraries was performed according to the Novaseq 6000 System User Guide (Part # 20023471) in a paired-end mode using a read length of 150 bp.

Supplementary tables:

Tables included as separate excel files:

Table S1: Metadata table for the 56 samples considered in this study. This table can be used with the reproducible workflow available on github. Details of columns: Trench system of origin, Depth zone/realm, Sampling site, Water depth at sampling site (m), Sediment core number, Bounds of sediment layer (cm), Geochemical zonation (defined in Schauberger *et al*. (1)), Unique site-core identifier and Simple sample label for convenience.

Table S2: Correspondence table between metagenome names and ENA information, read number statistics before and post-quality control, co-assembly group affiliation and percentage of reads mapping to the AOA MAGs considered in this study.

Table S3: Information for the 35 MAGs retrieved from other deep-sea studies.

Table S4: Statistics for the AOA MAGs reconstructed from abyssal and hadal surface sediments. Completeness and redundancy estimates were obtained from CheckM's lineage workflow (2).

Table S6: Co-assembly statistics for the 10 co-assembly groups defined in Table S2.

Table S5: Linear regression results for the evolution of the fixation index with shared SNV fractions.

		FST ~ Shared SNV positions (%)		FST ~ Shared SNV positions with same consensus (%)	
MAG name	Clade	adjusted R^2	p-value	adjusted R^2	p-value
HAK_Bin_00079	amoA-NP-gamma-2.1	0.9655	< 2.2e-16	0.9157	1.647e-13
HKT_Bin_00027	amoA-NP-gamma-2.1	0.8239	< 2.2e-16	0.1264	0.04932
H3T_Bin_00167	amoA-NP-gamma-2.1	0.8221	< 2.2e-16	0.5803	9.233e-06
AK7_Bin_00137	amoA-NP-gamma-2.1	0.7473	< 2.2e-16	0.3402	0.001646
H3D_Bin_00215	amoA-NP-gamma-2.1	0.7909	< 2.2e-16	0.6771	4.848e-07
HKT_Bin_00076	amoA-NP-gamma-2.1	0.7792	< 2.2e-16	0.8518	8.36e-11
HKT_Bin_00075	amoA-NP-gamma-2.1	0.848	< 2.2e-16	0.8798	6.308e-10
HAS_Bin_00039	amoA-NP-gamma-2.2	0.9223	6.666e-14	0.8148	9.931e-10
H3T_Bin_00024	amoA-NP-delta	0.439	0.0002512	0.3647	0.001058
A7D_Bin_00065	amoA-NP-delta	0.5711	1.181e-05	-0.0451	0.9315
A9S_Bin_00058	amoA-NP-delta	0.966	< 2.2e-16	0.8215	6.588e-10
A7D_Bin_00052	amoA-NP-delta	0.8851	5.024e-12	0.5943	6.301e-06
AK7_Bin_00037	amoA-NP-delta	0.7866	4.792e-09	0.9531	2.555e-16
AK7_Bin_00136	amoA-NP-delta	0.8791	8.77e-12	0.1441	0.03803
A7S_Bin_00119	amoA-NP-delta	0.4088	0.000459	0.1925	0.0184
A9S_Bin_00032	amoA-NP-theta	0.9676	4.553e-15	0.9618	2.018e-14
A7D_Bin_00152	amoA-NP-theta	0.9329	3.238e-12	0.9853	< 2.2e-16
A7S_Bin_00118	amoA-NP-theta	0.916	2.473e-11	0.6463	1.182e-05
AK7_Bin_00081	amoA-NP-theta	0.9795	3.872e-15	0.9811	2.013e-15
A7D_Bin_00162	amoA-NP-theta	0.9882	< 2.2e-16	0.9635	3.855e-13

Supplementary figures:

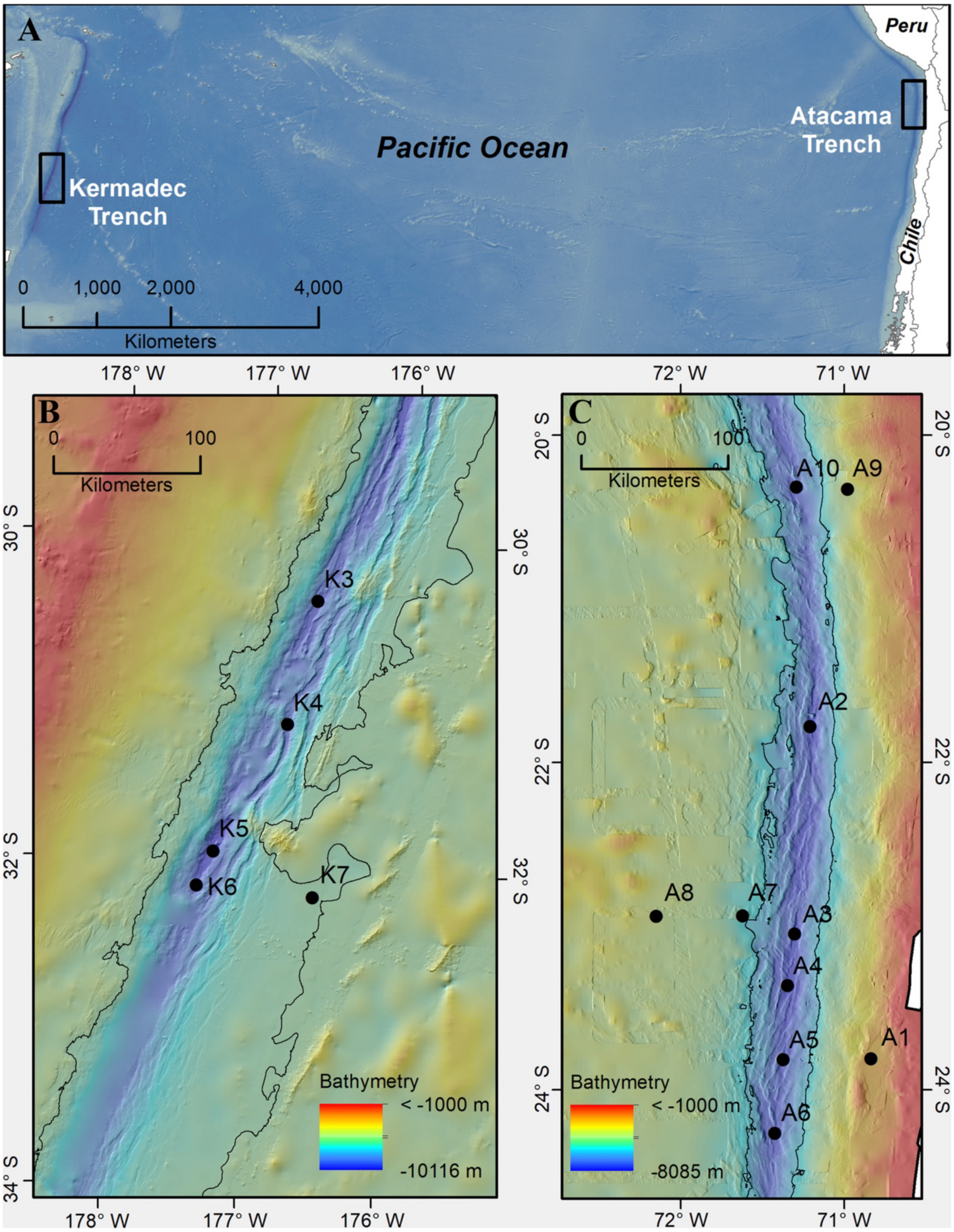

Figure S1: Maps of the sampled regions, extracted from Schauberger *et al.* (3). (**A**) Location of the trenches of interest (black boxes) in the South Pacific Ocean. Bathymetric maps with sampling sites (black circles) in the Kermadec Trench (**B**), and Atacama Trench (**C**). All bathymetry data were sourced from the Global Multi-Resolution Topography Synthesis (4)

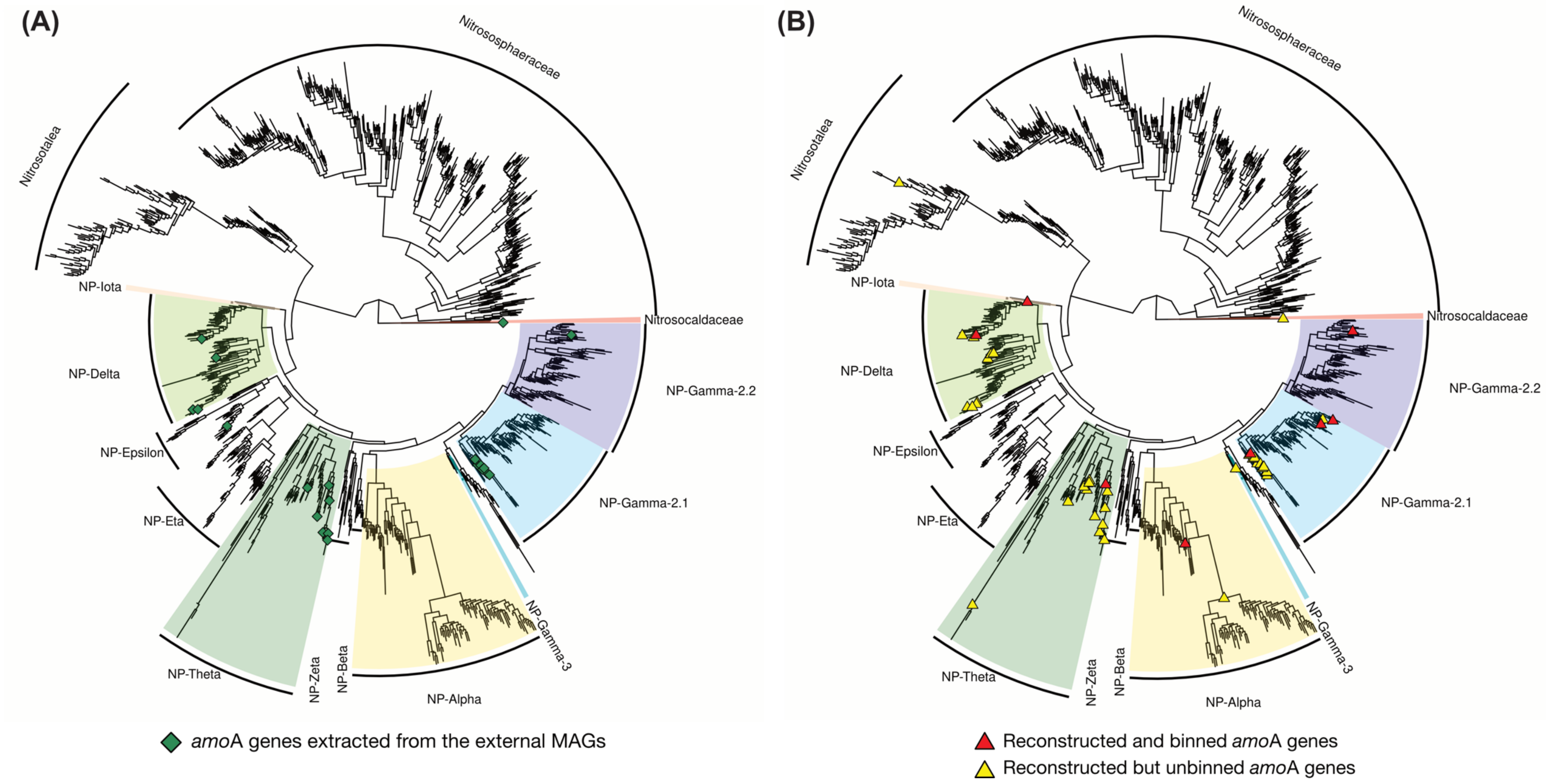

Figure S2: Phylogenetic tree of *amo*A genes obtained from Alves *et al*. (5), with placement of *amo*A genes extracted from external MAGs (green diamonds) **(A)**, and genes identified in co-assemblies from this study **(B).** Red triangles denote genes that were found in a MAG after binning, while yellow triangles illustrate genes that were unbinned. Shading highlights *amo*A clades as defined in Alves *et al.* (5).

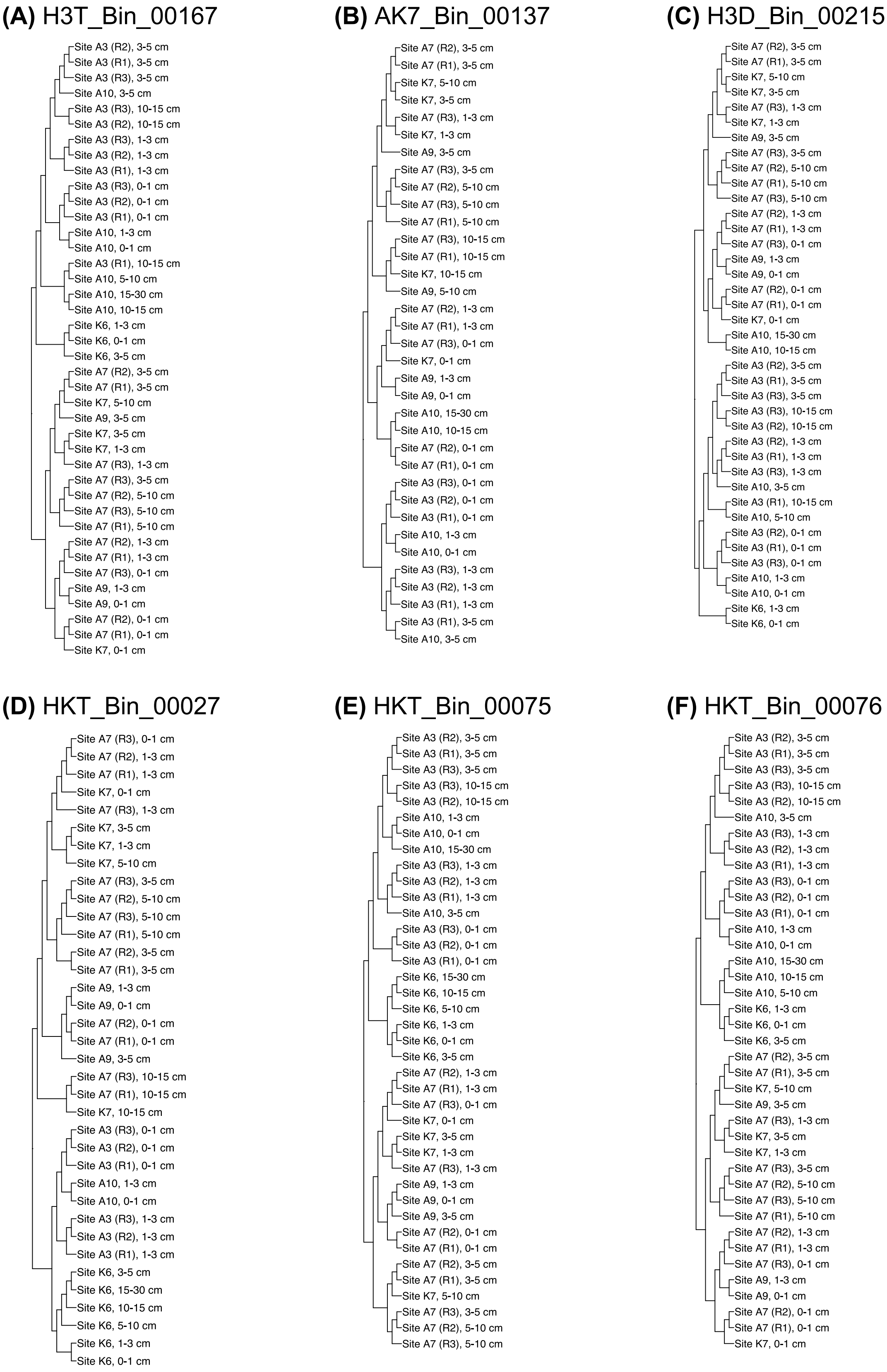

Figure S3: Clustering of samples based on the fixation index (F_{ST}) computed using nucleotide-level variability profiles for additional *amo*A-NP-gamma-2.1 MAGs.

(A) HKT_Bin_00022

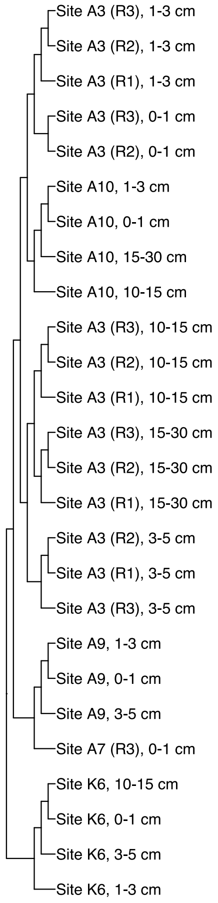

Figure S4: Clustering of samples based on the fixation index (F_{ST}) computed using nucleotide-level variability profiles for additional *amo*A-NP-gamma-2.2 MAGs.

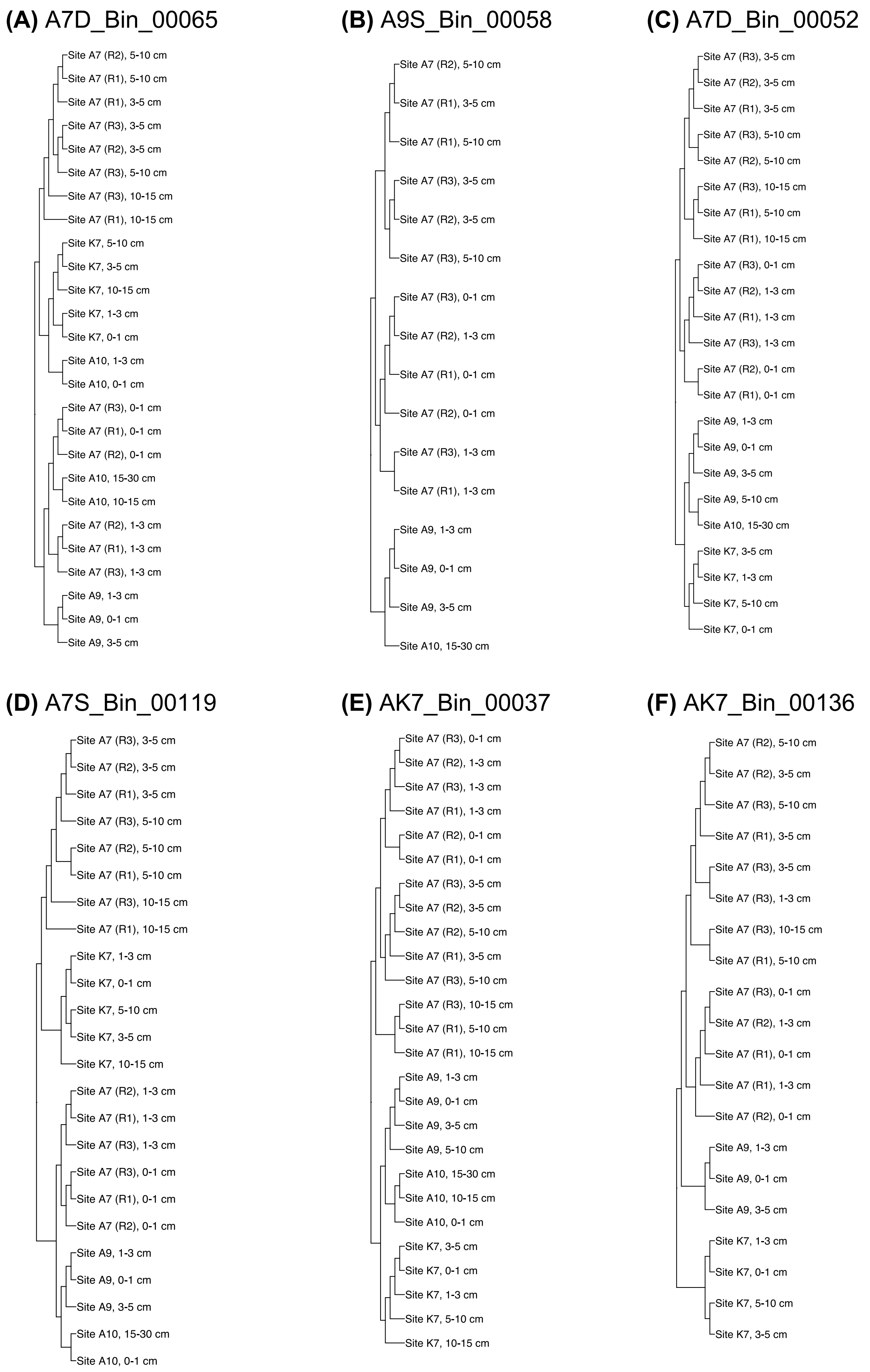

Figure S5: Clustering of samples based on the fixation index (F_{ST}) computed using nucleotide-level variability profiles for additional *amo*A-NP-delta MAGs.

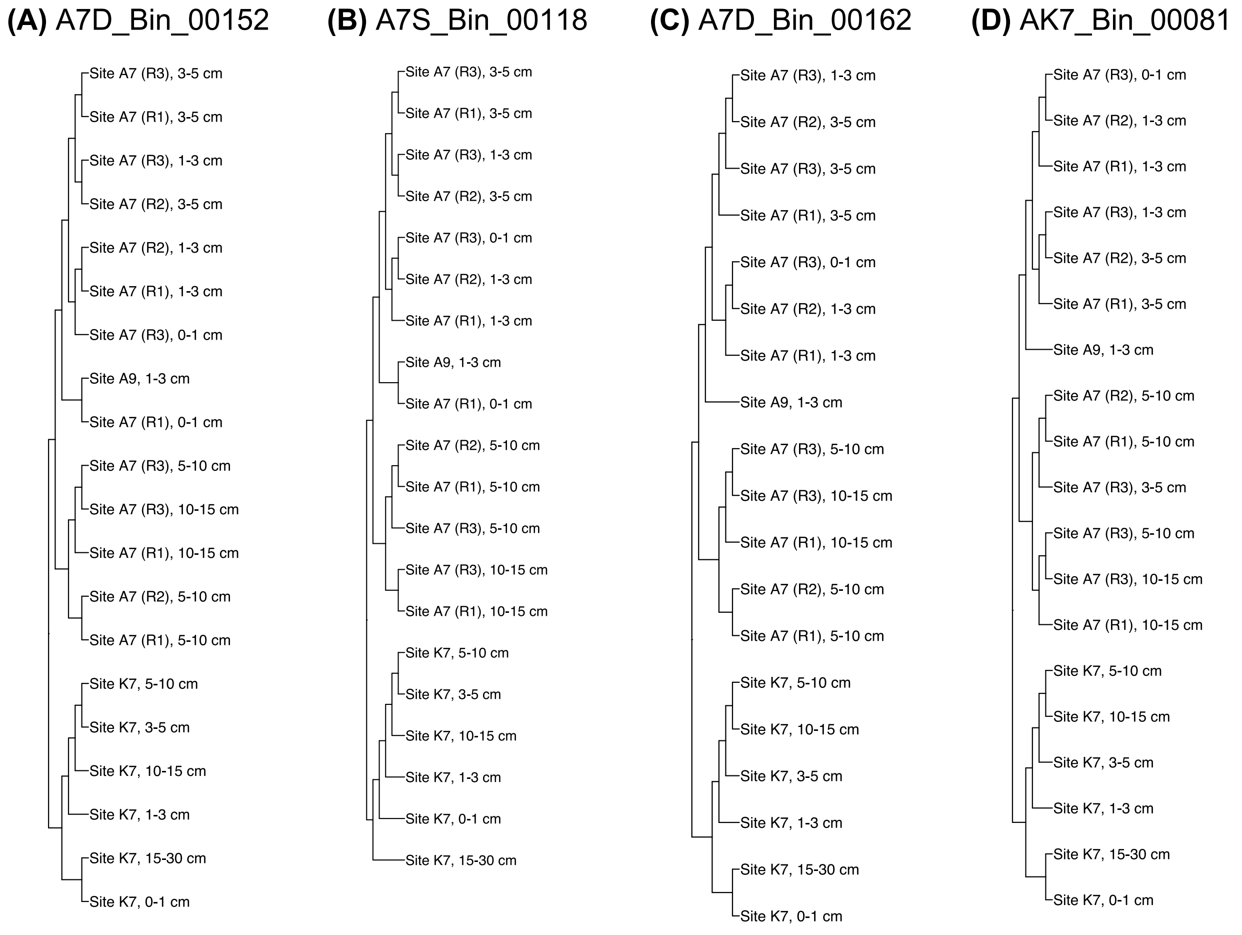

Figure S6: Clustering of samples based on the fixation index (F_{ST}) computed using nucleotide-level variability profiles for additional *amo*A-NP-theta MAGs.

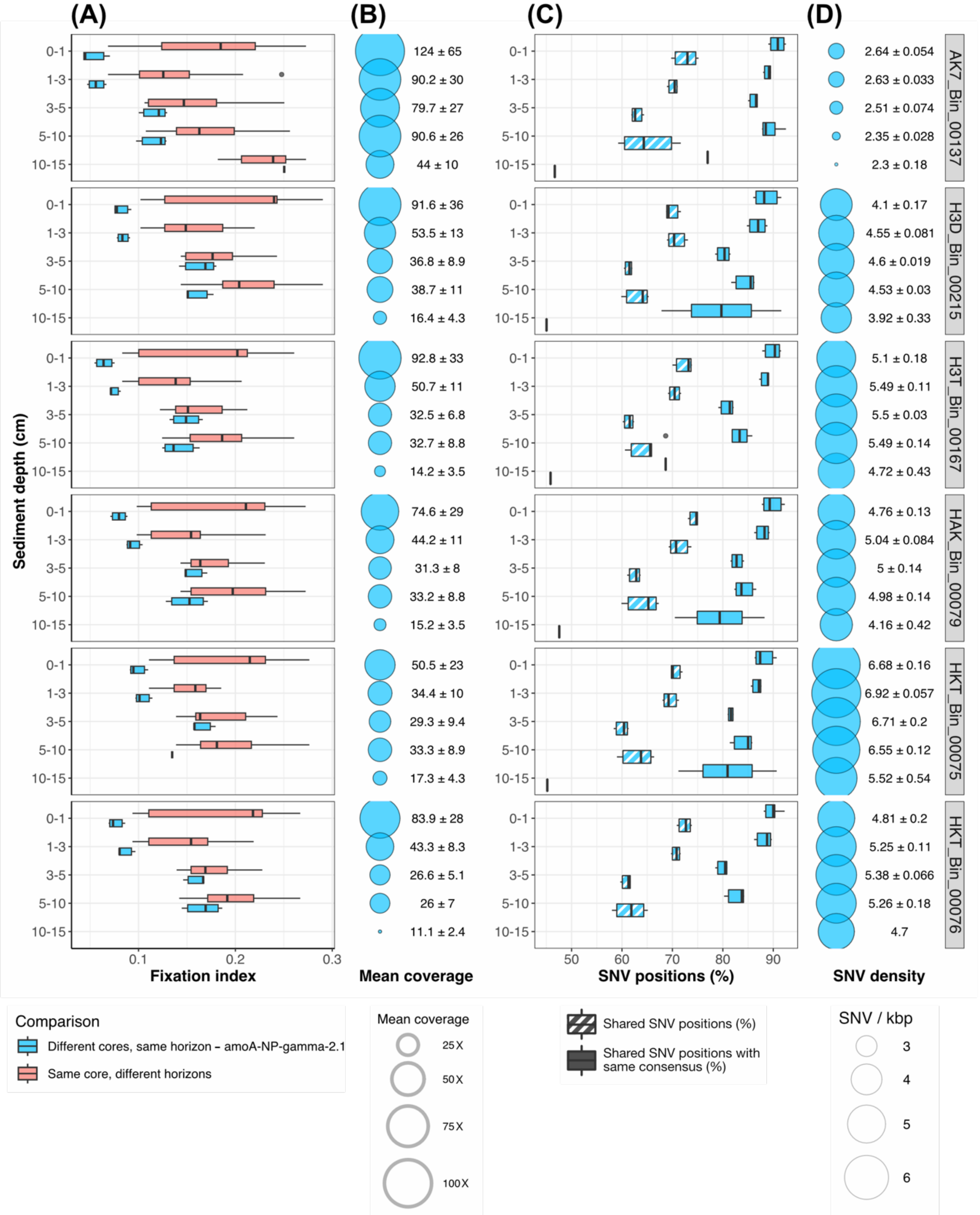

Figure S7: (A) Downcore comparison of fixation index (F_{ST}) values between core triplicates of abyssal site A7, for additional MAGs of clade *amo*A-NP-gamma-2.1. Comparison between the same sediment layers from different cores are in sky blue and comparison between different horizons of the same core in red. **(B)** MAG mean coverage in each sediment layer of the triplicate A7 cores, illustrated by circle size and clarified as mean ± standard deviation. **(C)** Percentage of single nucleotide variants (SNV) identified as variable positions in both samples when comparing similar sediment layers originating from different cores, and percentage of these shared SNVs presenting the same consensus nucleotide. **(D)** Mean density of SNVs in each sediment layer, summarized as mean ± standard deviation.

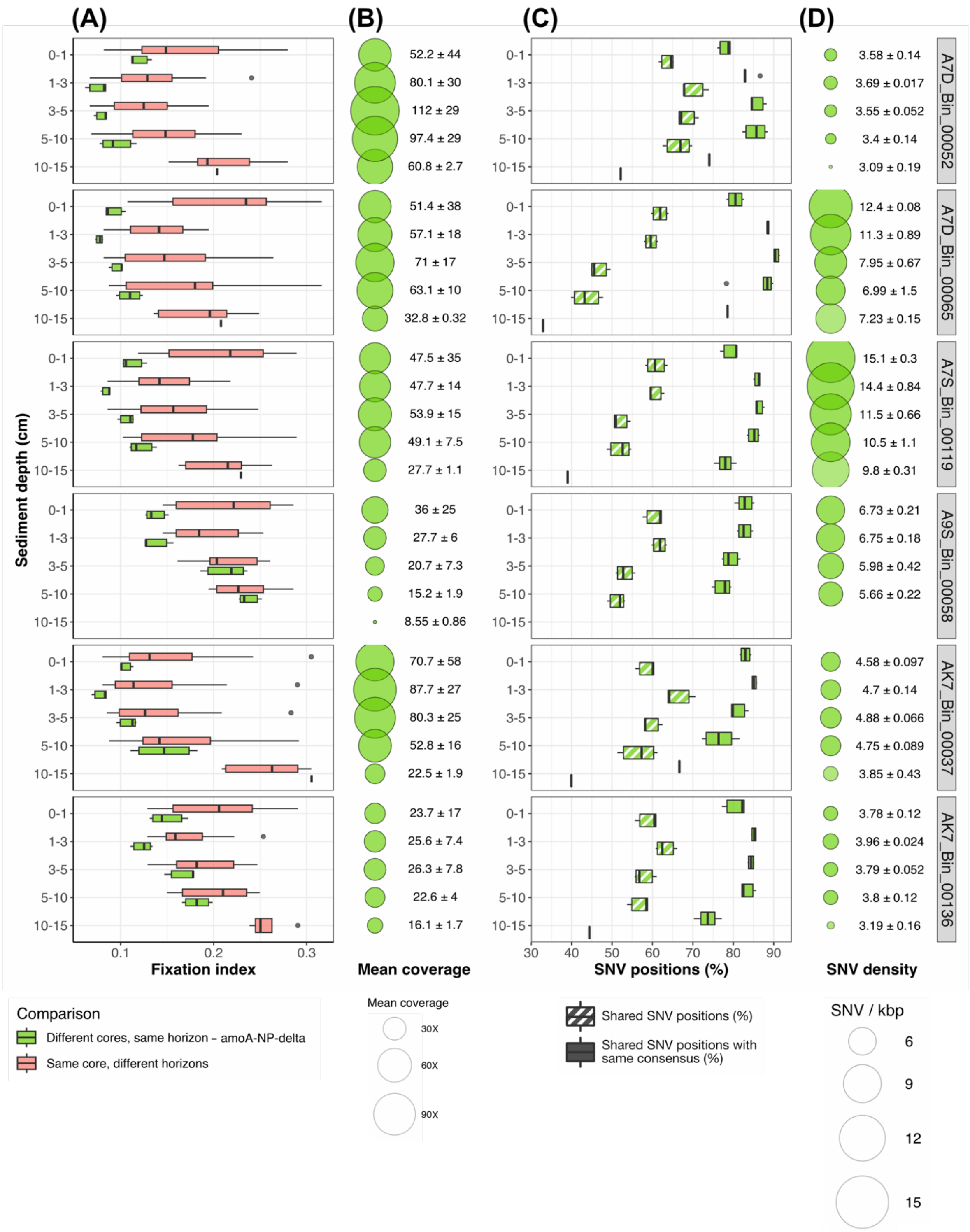

Figure S8: (A) Downcore comparison of fixation index (F_{ST}) values between core triplicates of abyssal site A7, for additional MAGs of clade *amo*A-NP-delta. Comparison between the same sediment layers from different cores are in bright green and comparison between different horizons of the same core in red. **(B)** MAG mean coverage in each sediment layer of the triplicate A7 cores, illustrated by circle size and clarified as mean ± standard deviation. **(C)** Percentage of single nucleotide variants (SNV) identified as variable positions in both samples when comparing similar sediment layers originating from different cores, and percentage of these shared SNVs presenting the same consensus nucleotide. **(D)** Mean density of SNVs in each sediment layer, summarized as mean ± standard deviation.

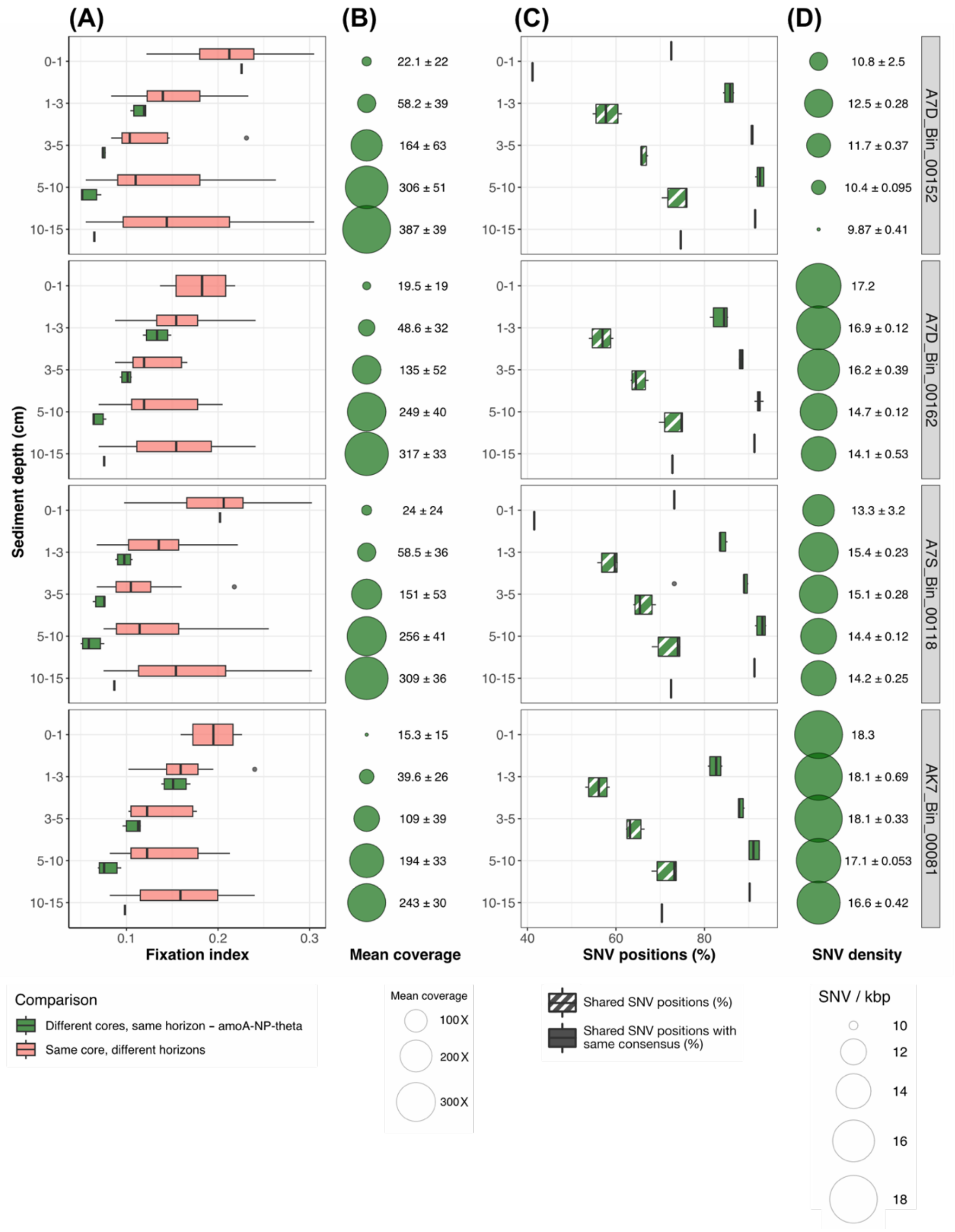

Figure S9: (A) Downcore comparison of fixation index (F_{ST}) values between core triplicates of abyssal site A7, for additional MAGs of clade *amo*A-NP-theta. Comparison between the same sediment layers from different cores are in dark green and comparison between different horizons of the same core in red. **(B)** MAG mean coverage in each sediment layer of the triplicate A7 cores, illustrated by circle size and clarified as mean ± standard deviation. **(C)** Percentage of single nucleotide variants (SNV) identified as variable positions in both samples when comparing similar sediment layers originating from different cores, and percentage of these shared SNVs presenting the same consensus nucleotide. **(D)** Mean density of SNVs in each sediment layer, summarized as mean ± standard deviation.

References:

1. Schauberger C, Glud RN, Hausmann B, Trouche B, Maignien L, Poulain J, et al. Microbial community structure in hadal sediments: high similarity along trench axes and strong changes along redox gradients. ISME J [Internet]. 2021 Jun 8 [cited 2021 Jun 8]; Available from: http://www.nature.com/articles/s41396-021-01021-w

2. Parks DH, Imelfort M, Skennerton CT, Hugenholtz P, Tyson GW. CheckM: assessing the quality of microbial genomes recovered from isolates, single cells, and metagenomes. Genome Res. 2015;25:1043–55.

3. Schauberger C, Middelboe M, Larsen M, Peoples LM, Bartlett DH, Kirpekar F, et al. Spatial variability of prokaryotic and viral abundances in the Kermadec and Atacama Trench regions. Limnol Oceanogr. 2021 Feb 28;lno.11711.

4. Ryan WBF, Carbotte SM, Coplan JO, O'Hara S, Melkonian A, Arko R, et al. Global Multi-Resolution Topography synthesis: GLOBAL MULTI-RESOLUTION TOPOGRAPHY SYNTHESIS. Geochem Geophys Geosystems. 2009 Mar;10(3):n/a-n/a.

5. Alves RJE, Minh BQ, Urich T. Unifying the global phylogeny and environmental distribution of ammonia-oxidising archaea based on amoA genes. Nat Commun. 2018;17.
